# VOLN27B: A New Head-Tailed Halovirus Isolated from an Underground Salt Crystal and Infecting *Halorubrum*

**DOI:** 10.1155/2021/8271899

**Published:** 2021-12-14

**Authors:** Shaoxing Chen, Yongpei Dai, Jingwen Liu, Shimin Zhang, Feilong Chen, Fanjie Jin, Peiyao Ruan, Lu Li, Xiangdong Chen

**Affiliations:** ^1^College of Life Sciences, Anhui Normal University, Wuhu 241000, China; ^2^Key Laboratory for Plant Diversity and Biogeography of East Asia, Kunming Institute of Botany, Chinese Academy of Sciences, Kunming 650201, China; ^3^State Key Laboratory of Virology, College of Life Sciences, Wuhan University, Wuhan 430072, China

## Abstract

A novel halovirus, VOLN27B, was isolated from a drill core sample taken at a depth of approximately 430 m, from a layer formed during the Cretaceous period (Anhui, China). VOLN27B infects the halophilic archaeon *Halorubrum* sp. LN27 and has a head-tailed morphotype with a contractile tail, typical of myoviruses. The average head diameter is 64 ± 2.0 nm, and uncontracted tails are 15 ± 1.0 × 65 ± 2.0 nm. The latent period is about 10 h. The maturing time of VOLN27B in cells of *Halorubrum* sp. LN27 was nearly 8 h. The adsorption time of VOLN27B on cells of *Halorubrum* sp. LN27 was less than 1 min. Virus particles are unstable at pH values less than 5 or when the NaCl concentration is below 12% (*w*/*v*). VOLN27B and *Halorubrum* sp. LN27 were recovered from the same hypersaline environment and provide a new virus-host system in haloarchaea.

## 1. Introduction

Hypersaline environments, near salt saturation, are commonly found to harbor members of the extremely halophilic archaea (haloarchaea; Class *Halobacteria*), which are often the dominant cellular microorganisms and can grow to high cell densities (>10^7^ cells per milliliter). Viruses infecting haloarchaea are typically observed to be in much higher concentration than their host cell population, and direct examination of natural brines shows a number of different morphological types of virus-like particles (VLP), including spherical, spindle-shaped, and head-tail [1–4]. In a comprehensive study of a salt lake in Senegal (Lake Retba), the morphotypes (and percentage) of VLPs were as follows: spindle-shaped/fusiform (43%), spherical (35%) particles, and head-tail (1%), while linear and various other, unusual morphological particles accounted for the remainder [3]. Pleomorphic virus particles are too difficult to distinguish in direct negative-stain EM of natural waters and do not get counted. Since grazing protists are usually absent at saturating salt conditions, the high abundance of viruses indicates their important role as predators [5], making them not only major drivers of haloarchaeal evolution but also significant players in biogeochemical cycling [6]. Although head-tailed VLPs are a less-frequent morphotype observed in hypersaline environments, they are commonly isolated and show great diversity [7]. For example, in 1974, the first haloarchaeal virus (and the first archaeal virus) to be described was the myovirus Hs1 [8]. Nowadays, a brand new taxonomy of archaeal tailed viruses has been proposed that all genome-sequenced archaeal tailed viruses were classified into three orders, i.e., *Thumleimavirales*, *Kirjokansivirales*, and *Methanobavirales* (https://talk.ictvonline.org/files/proposals/archaeal-viruses/m/ec-approved-awaiting-ictv-ratification).

Since 1974, more than 70 head-tailed viruses infecting haloarchaea have been reported, and their morphotypes include those with contractile tails (myovirus), noncontractile tails (siphovirus), and very short tails (podovirus) [3, 7, 9–11]. Relatively few have had their genome sequenced [3, 11–16]. Recently, Liu et al. and colleagues reported 37 new genomes of haloarchaeal tailed viruses and proposed 14 new families (https://talk.ictvonline.org/files/proposals/archaeal-viruses/m/ec-approved-awaiting-ictv-ratification). Haloarchaeal viruses Hardycor1, Hardycor2, and Serpecor1 are able to infect *Halorubrum coriense* strain Ch2, while ChaoS9 and HFTV1 can infect *Halobacterium salinarum* and *Haloferax* strains, respectively. HFTV1 can also infect *Halorubrum* spp. although it is eight times less effective than *Haloferax* strains [17].

To date, more than 100 viruses have been isolated from hypersaline environments, a majority of them (~90) infect extremely halophilic euryarchaea in the class Halobacteria [3, 10]. All the cultivated haloarchaeal viruses are categorized into four different morphological groups: spindle-shaped (family *Halspiviridae*), pleomorphic (family *Pleolipoviridae*), tailless icosahedral (family *Spherolipoviridae*), and tailed icosahedral (order *Thumleimavirales*, formerly named order *Caudovirales*) [10, 18]. Archaea are infected by a variety of morphologically diverse viruses [19]. The diversity of archaeal viruses (~150) is severely underrepresented compared with that of viruses infecting bacteria and eukaryotes (>6000), limiting our understanding of their evolution and environmental impacts [3].

In this study, we isolated a new infectious head-tailed virus, namely, VOLN27B, infecting *Halorubrum* sp. LN27 from a drill-core of salt mine (~430 m underground) which formed during the Cretaceous period (Anhui, China). Here, we determined the primary viral characteristics, including particle morphology, stability, virus life cycle, and nucleic acid type. This provides the first description of a novel virus-host system recovered from an extremely hypersaline environment that can be applied to studies of virus-haloarchaea interactions and coevolution.

## 2. Materials and Methods

### 2.1. Sample Collection, Culture Medium, and Growth Conditions

**Figure 1:**
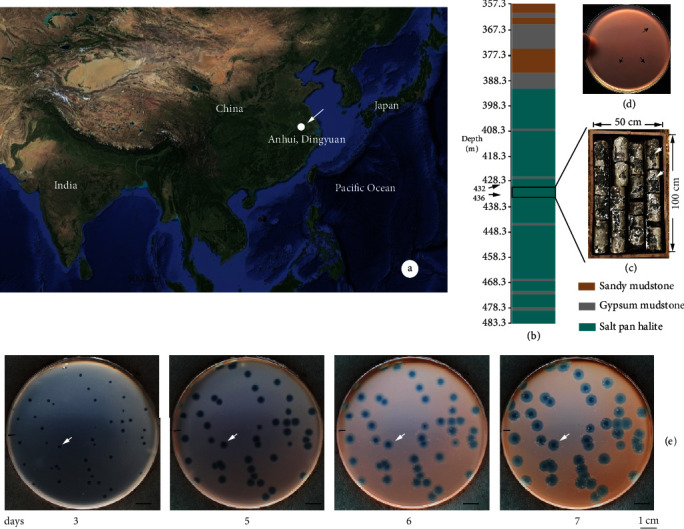
Sampling and halovirus screening. The drill core was collected from Dingyuan Salt Mine, Anhui, China (white arrow) (a). Schematic map showed the stratigraphic texture of the drill core sample. The bottom of the drill core reached 483.3 m subsurface (b). The layers constituted of salt crystals were colored in indigo blue. A section of the salt layer from 432 to 436 m underground was taken from drill core sample. Two sections of drill core almost consisted of salt crystals (white arrows) were used for screening haloarchaea and halovirus (c). Plaques were formed on the lawn of strain *Halorubrum* sp. LN27 after cultivation for 3 days at 37°C (d). Clear and transparent plaques can be easily recognized after a cultivation of 3 days. As the extension of the cultivation time, the diameter of the plaque became larger and arrived approximately 7 mm after 7 days. Black arrow showed the morphologic development of a single plaque (e).

A drill core was collected at the Dingyuan Salt Mine (longitude 117.527; latitude 32.508), Anhui province, China, in May 2016 (Figure 1). The entire core was about 500 m long, contained several layers of salt and rock, and all sections were completely sealed with wax to insulation them against entrance of atmosphere and moisture loss. Two sections of drill core, at approximately 400 m depth, consisted of salt crystals and were unwaxed and washed with HCl (10 M) and NaOH (10 M) three times each to remove surface contamination. The salt was then dissolved in about 20 L sterile salt water (5% NaCl, *w*/*v*) after washing with distilled water for six times to entirely scour off the residual NaOH. Insoluble substances were removed by suction filtration with sterile filter paper (GE Whatman, 3MM). The filtrate was concentrated from about 22 L to 500 mL through tangential flow filtration (TFF) using Vivaflow 50 (Sartorius, Germany) with a molecular weight cut-off (MWCO) at 10 kDa. The concentrated salt solution was stored in a refrigerator at 4°C until halovirus screening.

The haloarchaea used as host strains to isolate haloviruses are listed in Table S1, and the additional strains used for probing host range are listed in Table S2. All strains were cultivated aerobically at 37°C using JCM 168 medium (https://www.jcm.riken.jp/) (pH 7.5). The medium consisted of Casamino acids (BD-Difco) 5.0 g, yeast extract (BD-Difco) 5.0 g, sodium glutamate 1.0 g, trisodium citrate 3.0 g, MgSO_4_·7H_2_O 20.0 g, KCl 2.0 g, NaCl 200.0 g, FeCl_2_·4H_2_O 36.0 mg, and MnCl_2_·4H_2_O 0.36 mg per liter. Solid and soft agar (top layer) media were obtained by adding 15 g or 5 g of Bacto agar (Difco Laboratories) per liter, respectively.

### 2.2. Virus Isolation and Host-Range Detection

To screen for haloviruses present in the concentrated salt solution obtained from the drill core sample, plaque assays were performed on ten haloarchaeal strains (Table S1). Three were autochthonous strains of *Halorubrum* that have been previously described [20], and the rest were culture collection strains belonging to seven different genera. A 100 *μ*L volume of the salt solution was mixed with 900 *μ*L of cell suspension (exponential phase, OD600 ≈ 1.5) and incubated at 37°C for 30 min; then, the entire mixture was added to 5 mL soft agar (50°C, JCM 168 medium), vortexed briefly, and poured on to a base layer agar plate (1.5% agar) of the same medium. Plates were incubated at 37°C for three days and observed for plaque formation. Several clear plaques were formed on the lawn of *Halorubrum* sp. strain LN27. Three consecutive plaque purifications were performed to obtain a pure culture. Ultimately, the halovirus infecting cells of strain LN27 was named VOLN27B.

Plaques on the top soft agar were picked with a sterile toothpick into JCM 168 liquid medium, homogenized by votexing, and retitrated on *Halorubrum* sp. strain LN27 using the plaque formation assay. To obtain a virus stock, titration plates with near confluent plaques were flooded with NaCl (20%, *w*/*v*) and followed by collecting the NaCl solution after incubation for about 12 h. The fluid collected from the plate was defined as primary virus stock (PVS). Virus titer in this PVS was determined to be approximately 10^11^ pfu/mL via plaque formation assay.

The influence of time and temperature on plaque formation was examined by titration of virus on lawns of strain LN27 and incubation of plates for (1) 3, 5, 6, and 7 days at 37°C and (2) incubation at 30°C, 38°C, 42°C, and 50°C for 3 days.

Host range was determined by testing the ability of each PVS to infect 25 different haloarchaeal strains, including 12 species of *Halorubrum*, two species of *Halopenitus*, two species of *Haloferax*, and one species from the following genera: *Halalkalicoccus*, *Halococcus*, *Haloarchaeobius*, *Halostagnicola*, *Haloterrigena*, *Haloparvum*, *Natrinema*, *Natronomonas*, and *Natrialba* (Table S2).

### 2.3. Phylogenetic Analysis of the *Halorubrum* sp. Strain LN27

Strain LN27 was isolated from the Dingyuan Salt Mine. Based on the sequence similarity of 16S rRNA gene, strain LN27 was classified as *Halorubrum* sp. LN27 [20]. To investigate the phylogenetic position of this strain within genus *Halorubrum*, the almost complete 16S rRNA gene sequence was obtained using the method described by Sun et al. [21]. Reference 16S rRNA gene sequences were retrieved from GenBank (https://www.ncbi.nlm.nih.gov/nucleotide/). Multiple sequence alignment and phylogenetic analysis were conducted by using the MEGA X (bootstrap, 1,000 replicates, Neighbor-joining) [22].

### 2.4. Virus Stability Assay

PVS was used for testing virus stability under different conditions, after which the virus titer was determined by plaque assay. Temperature stability was tested at (1) 4°C for 1, 2, 3, and 5 months; (2) 37, 50, 60, 70, 80, and 90°C for 10 min; and (3) frozen at -20°C, -40°C, and -70°C overnight followed by a thawing. Stability to pH was tested by diluting PVS 1 : 100 (*v*/*v*) in buffered 20% (*w*/*v*) NaCl solution adjusted to pH 4.0, 5.0, 6.0, 7.0, 8.0, 9.0, and 10.0 and incubation at room temperature for 12 h. The buffers used were pH 4.0 and 5.0, 2-morpholinoethanesulfonic acid; pH 6.0, 7.0 and 8.0, 1,4-piperazine bis-ethanesulfonic acid; pH 9.0, 2-cyclohexylamino ethanesulfonic acid; and pH 10.0, N-cyclohexyl-3-aminopropanesulfonic acid. All buffers were at 1 mol/L.

Salt stability was tested at by diluting PVS 1 : 100 (*v*/*v*) in 1, 2, 4, 8, 9, 10, 11, and 12% (*w*/*v*) NaCl solution and incubation at room temperature for 12 h. Chloroform sensitivity was tested by mixing PVS with an equal volume of chloroform (1 : 1, *v*/*v*), separation of the phases by centrifugation (12,000 rpm) at 4°C for 3 min, and removing the aqueous phase.

### 2.5. Virus Adsorption Time

To investigate the adsorption time, exponentially growing cells of *Halorubrum* sp. strain LN27 (host) and *Halorubrum* sp. strain LN60 (not host) were infected with halovirus VOLN27B (m.o.i 1 : 10) at postinfection (p.i.) times of 1, 5, 10, 15, 20, 25, and 30 min. The cells were removed from the sample through centrifugation (12,000 rpm) at 4°C for 10 min. Then, cell-free supernatants were used for plaque formation assay.

### 2.6. One-Step Growth Curve

A one-step growth curve was conducted in a 250 mL flask containing 100 mL liquid JCM 168 medium as follows: (1) Cells of strain LN27 were inoculated into the liquid medium and cultivated at 37°C to exponential phase (OD_600_ = 0.7 ~ 0.8; ≈2.0 × 10^8^ cfu/mL). (2) VOLN27B stock (10^11^ plaque forming unit per milliliter, pfu/mL) was added into above cell suspension to an m.o.i = 20. (3) After an adsorption period (30 min), virus infected cells were harvested by centrifugation (8,000 rpm, 10 min, 4°C) and washed gently with JCM 168 broth twice to remove unbound virus. Then, cells were resuspended with 100 mL JCM 168 broth; (4) the cell suspension was incubated at 37°C with shaking (180 rpm). The OD_600_ was measured in 1, 3, or 6 h intervals for the infected and uninfected cultures, respectively. (5) Viable cells in both infected and uninfected cultures were monitored using plate counts. The virus titer of the culture supernatants was determined by plaque formation assay.

Cells of *Halorubrum* sp. strain LN27 were grown to exponential phase (OD_600_ = 0.8), infected with halovirus VOLN27B (m.o.i = 20; p.i = 15 min), and unabsorbed virus removed by washing cells three times with 100 mL sterilized 20% NaCl solution. After resuspension in 100 mL JCM 168 broth, cells were incubated at 37°C with shaking (180 rpm). At hourly intervals, 100 *μ*L culture was removed, the cells collected by centrifugation (12,000 rpm, 3 min, 4°C), washed three times with 20% NaCl solution, lysed by the addition of 500 *μ*L ddH_2_O, and the virus titer of the lysate determined by plaque titration.

### 2.7. Determination of the Virus Genome Type

To determine the genome type of halovirus VOLN27B, the genome of VOLN27B was isolated from the virus stock according to the method described by Summer [23] using Wizard DNA Clean-Up System (Promega, USA). Genomic DNA (1 *μ*g) was digested with DNase I (5 U) (TaKaRa, Japan), Exonuclease III (20 U) (TaKaRa, Japan), and Mung Bean Nuclease (4 U) (TaKaRa, Japan) at 37°C for 1 h, and the resulting product was checked by DNA electrophoresis. An equal amount of undigested plasmid pBR322 (NEB, USA) was used as a circular dsDNA standard, and the EcoRI (TaKaRa, Japan) cleaved pBR322 was used as a linear dsDNA standard. These untreated and EcoRI leaved pBR322 plasmids were also used as controls in treatments nucleases DNAse I, Exonuclease III, and Mung Bean nuclease.

### 2.8. Virus Purification

*Halorubrum* sp. strain LN27 was grown in liquid cultures to midexponential phase (OD_600_ = 0.7, 100 mL) then infected with virus halovirus VOLN27B (m.o.i = 20) and incubated at 37°C with shaking (180 rpm). At 36 h p.i., the cells were removed by centrifugation at 8,000 rpm for 15 min at 4°C, and virus in the supernatant concentrated 10-fold using tangential flow filtration (TFF) with a molecular weight cut-off (MWCO) of 10 kDa. The concentrated virus (10^9^ pfu/mL) was precipitated using 10% (*w*/*v*) polyethylene glycol 6000 (PEG 6000) overnight at 4°C. Precipitated virus was collected by centrifugation (12,000 rpm, 15 min at 4°C), resuspended in 3 mL 20% (*w*/*v*) NaCl solution, loaded on to a linear 5% to 20% (*w*/*v*) sucrose gradient, and centrifuged at 28,000 rpm for 8 h at 4°C (Beckman OptimaXE-100 with SW40Ti rotor, USA). The virus zone was collected and stored at 4°C until use. The titer was ~10^11^ pfu/mL.

### 2.9. Electron Microscopy

To determine the morphology of halovirus VOLN27B, concentrated and purified virus particles were stained with 3% (*w*/*v*) uranyl acetate (pH 4.5) for 30 s, and the dried copper grid was subjected to observation through transmission electron microscope (TEM). Electron micrographs were taken using JEM-1400 Transmission Electron Microscope (80 kV) (JEOL, Japan).

## 3. Results

### 3.1. A New Halovirus Isolated from a Drill Core Salt Layer

A drill core sample from approximately 400 m underground was acquired from the Dingyuan Salt Mine (Anhui, China) (Figures 1(a)–1(c)). The area of the Dingyuan Salt Mine was formed during the Cretaceous period (145.5 to 65.5 million years ago) and has not been disturbed by transgressive events [24]. Ten strains representing different genera, i.e., *Halorubrum*, *Haloarcula*, *Halobaculum*, *Halococcus*, *Halalkalicoccus*, and *Halobellus*, including three strains isolated from the Dingyuan Salt Mine were used as host cells to screen for infectious haloviruses released from the dissolved halite of the drill core sample (Table S1). Plaques only formed on a lawn of *Halorubrum* sp. strain LN27 (Figure 1(d)). This halovirus infected *Halorubrum* sp. strain LN27 was denoted as VOLN27B. After 3 days of cultivation, plaques were presented clearly, and the boundary was transparent (Figure 1(d)). Regardless of the efficiency of infection, there were approximately 10 infectious haloviruses that infects *Halorubrum* sp. strain LN27 per one gram of salt mine sample from Dingyuan Salt Mine (China). With an extension of cultivation period, the diameter of plaques increased from 2-3 mm to 7-8 mm (Figure 1(e)).

### 3.2. Phylogenetic Position of the Host Strain LN27

In a previous study [20], the near complete 16S rRNA gene sequence of the haloarchaeal host strain LN27 was determined (1453 nt, accession MN829451.1). This was aligned to the sequences of closely related strains, such as *Halorubrum saccharovorum* strain JCM 8865 (99.31%, NR_113484.1) and the alignment used to reconstruct a phylogenetic tree (Fig. S1). The sequence of strain LN27 clusters within a major clade of *Halorubrum* species, branching most closely with *Hrr. saccharovorum* JCM 8865^T^ and *Hrr. persicum* C49^T^ (Fig. S1), and with good bootstrap support. This isolate was therefore designated *Halorubrum* sp. strain LN27.

### 3.3. VOLN27B Has a Narrow Host Range

To probe the host range of VOLN27B, plaque assays were performed with twenty-five haloarchaeal strains from twelve genera, including twelve closely related type strains from the genus *Halorubrum* (Table S2). The virus only produced plaques on lawn of *Halorubrum* sp. LN27, indicating a relatively narrow host range.

### 3.4. Stability of Halovirus VOLN27B

**Figure 2:**
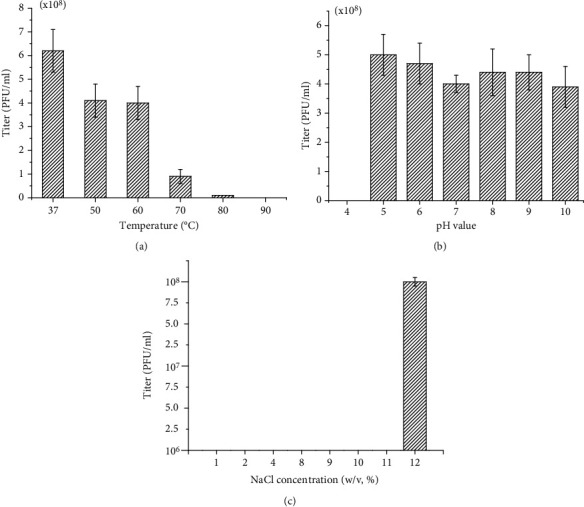
Stability of VOLN27B under different temperature, pH, and salt concentration. Halovirus VOLN27B was heated by different temperature for 10 min prior to conducting plaque formation assay. Virus titer decreased along with the increase of temperature. Viruses were inactivated at 90°C (a). When VOLN27B was kept in NaCl solution with different pH for 12 h, the infectivity was largely blocked when the pH below 5. VOLN27B was relatively stable in alkaline condition (b). VOLN27B was unstable in NaCl solution with the concentration below 12% overnight (*w*/*v*) (c).

The stability of VOLN27B was tested under various conditions, and the results are summarized in Figure 2. Heating to 80°C for 10 min, virus titer still has 10^7^ pfu/mL, suggesting that VOLN27B is relatively stable for which it can retain some of infectivity in a high-temperature condition albeit a reduction of virus titer in three orders of magnitude. But we did not obtain any plaques after a heat treatment at 90°C for 10 min (Figure 2(a)). VOLN27B is relatively stable in alkaline condition, even in pH 10.0, whereas virus titer decreased sharply when pH < 5.0 (Figure 2(b)). VOLN27B can survive well in a condition with no less than 12% (*w*/*v*) of NaCl concentration (Figure 2(c)). Most of infectious viruses isolated from hypersaline environment infecting haloarchaea were salt-dependent [25].

**Figure 3:**
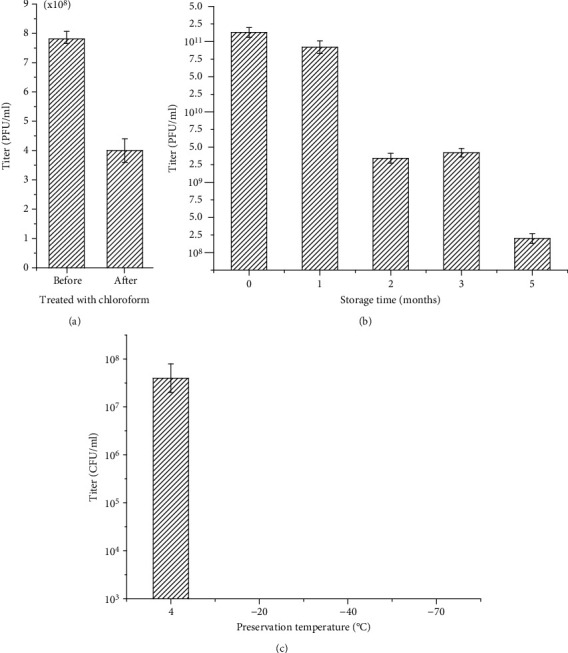
Influence of chloroform extraction, preservation temperature, and storage time on virus titer. Chloroform extraction slightly reduced virus titer but not in order of magnitude (a). With passage of time from 1 month to 5 months at 4°C, the titer of halovirus VOLN27B in cell-free supernatants decreased from ~10^11^ to ~10^8^ pfu/mL (b). Virion particles preserved in 4°C were much more stable than in minus 20, 40, and 70°C (c).

Chloroform treatment reduced the titer by half (Figure 3(a)), suggesting that VOLN27B does not contain a lipid membrane. After storage of halovirus VOLN27B at 4°C for five months, the virus titer decreased from 10^11^ to 10^8^ pfu/mL (Figure 3(b)). Freezing virus overnight at -20°C, -40°C, and -70°C, abolished infectivity (Figure 3(c)). The formation of ice crystals may cause devastating damage to virion particles.

**Figure 4:**
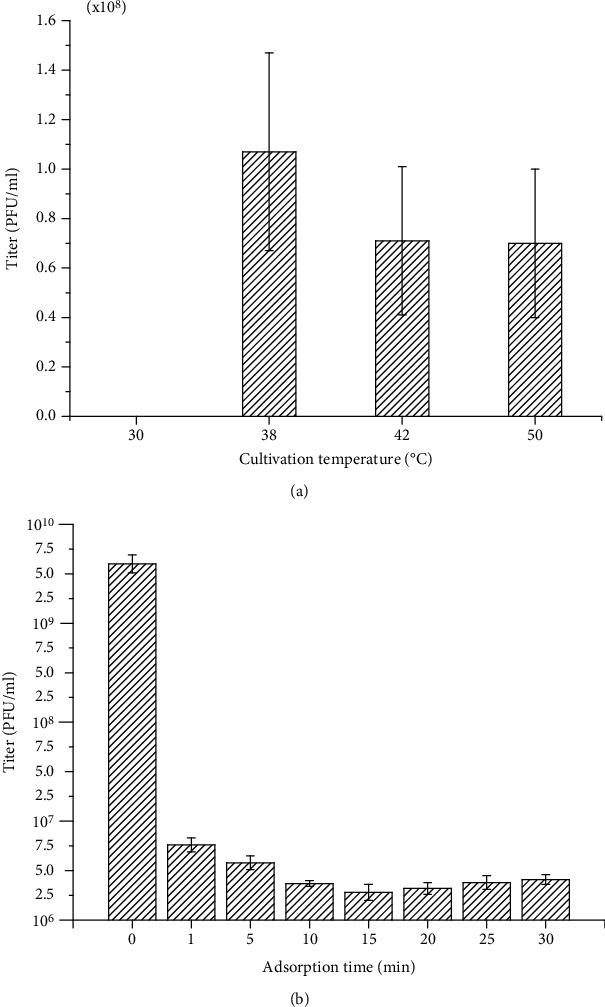
Influence of cultivation temperature on virus stability and its adsorption time. To determine whether VOLN27B is an enveloped virus, virus stock was resuspended with equal volume of chloroform (1 : 1; *v*/*v*), then performed the plaque formation assay. Cultivation temperature after infection would impact the production of viruses. No visible plaques were observed when cells of strain *Halorubrum* sp. LN27 were cultivated at 30°C or lower (a). The adsorption time for VOLN27B to its host, *Halorubrum* sp. LN27, was less than 1 min (m.o.i = 1/10) (b).

When the host strain of VOLN27B, *Halorubrum* sp. LN27, was cultivated in an incubator with a temperature lower than 38°C, plaques can be hardly recognized after three days of cultivation. However, plaques on a lawn of *Halorubrum* sp. LN27 can be easily recognized even when the *Halorubrum* sp. LN27 infected by VOLN27B was cultivated at 50°C (Figure 4(a)).

Compared to haloviruses S5100 and S50.2 which infect *Halobacterium cutirubrum* [26], the VOLN27B presented a very short time for adsorption. There was no significant difference in virus titer in supernatants after 1 min contact when m.o.i is 1/10, indicating that the vast majority of VOLN27B can rapidly absorb onto the cell surface or enter into cells of strain LN27 within 1 min (Figure 4(b)).

### 3.5. VOLN27B Is a Lytic Halovirus

**Figure 5:**
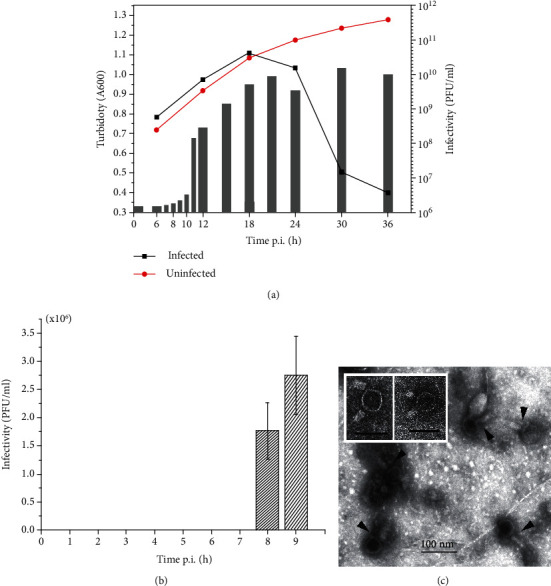
One-step-growth-curve of VOLN27B infecting cells of strain *Halorubrum* sp. LN27 and of morphotype VOLN27B virions. Growth of uninfected (red line with filled red circle) and virus infected (m.o.i. = 20) (black line with filled black square) *Halorubrum* sp. LN27 was determined by measuring the absorbance at 600 nm (*A*_600_) (a). Virus titer in virus infected *Halorubrum* sp. LN27 supernatant was determined by plaque formation assay using *Halorubrum* sp. LN27 as lawn cells (gray bars). A significant production of viruses started at 10 h postinfection (p.i.). And at 18 h p.i., the turbidity of cell suspension began to decline significantly. At 21 h p.i., the virus titer reached the maximum (a). To determining the maturing time for VOLN27B in cells of strain *Halorubrum* sp. LN27, the free viruses were removed by washing with sterilized 20% NaCl solution for three times after conducting the infection. Cells collected from 100 *μ*L cell suspension by centrifugation (12,000 rpm. 3 min, 4°C) were lysed with sterilized ddH_2_O (500 *μ*L); then, the lysates were used to perform plaque formation assay immediately. Infectious viruses were detected at 8 h p.i. (b). Morphotype of VOLN27B was determined by using transmission electron micrograph (JEM-1400, JEOL, Japan) after staining with 3% (*w*/*v*) uranyl acetate (pH 4.5). Arrowheads show the intact virus particles. Scale bar, 100 nm (c).

Turbidity of cell suspension infected by VOLN27B (m.o.i = 20) begun to drop at 18 h p.i (hour postinfection) (Figure 5(a)). Although the turbidity at OD_600_ decreased sharply and reached the lowest at 36 h p.i, viable cells in these virus supernatants were still remained (data no shown). A significant production of free viruses in supernatants began at 10 h p.i. and peaked at approximately 21 h p.i. (Figure 5(a)). The sharp decline in cell density accompanied by virus production illustrated that virus multiplication lead to cell lysis.

When cells of *Halorubrum* sp. LN27 were infected by VOLN27B, no infectious viruses were detected before 8 h p.i (Figure 5(b)), suggesting that VOLN27B required at least 8 h for the maturing of halovirus VOLN27B from adsorption to production of mature virus particles. The latent period of halovirus VOLN27B in cells of *Halorubrum* sp. LN27 was approximately 10 h (Figure 5(b)).

### 3.6. Halovirus VOLN27B Has a Head-Tailed Morphotype

Negative-stain electron microscopy of highly purified VOLN27B revealed head-tailed particles, some with contracted tails (Figure 5(c)), which was morphologically similar to the HF1-group haloviruses [13, 27]. The average dimension of VOLN27B was determined based on calculation of 20 well-preserved virion particles. VOLN27B has a typical icosahedral head (64 ± 2.0 nm) and a long tail (15 ± 1.0 × 65 ± 2.0 nm). The size of the tail sheath is approximately 20 ± 0.5 × 35 ± 1.0 nm. The morphotype of VOLN27B is that of a typical myovirus.

### 3.7. The VOLN27B Genome Is a Linear Double-Stranded DNA Molecule

DNA extracted from purified VOLN27B could be digested with DNase I and Exonuclease III but not by Mung Bean Nuclease (Fig. S2a), indicating that the viral genome is dsDNA. Plasmid pBR322 was used as a control substrate to confirm the specificity of each of the three nucleases (Fig. S2 b and c).

## 4. Discussion

Hypersaline environments, such as salt lakes, solar salterns, and salt mines, are categorized into two types, athalassohaline and thalassohaline, based on comparison of the elemental composition to the sea water. Dingyuan Salt Mine (Anhui, China) is an inland athalassohaline hypersaline environment formed approximately 650 m.y.a (millions of years ago) without any marine transgressions [24]. *Halorubrum* sp. strain LN27 was isolated during the exploration of microbial diversity in the Dingyuan Salt Mine through a culture-dependent approach [20]. Later, the microbial community of this salt mine has been revealed systematically via high-throughput sequencing [23]. Numerous researches have proved that hypersaline environments are dominated by haloarchaea, and viruses outnumbered haloarchaea in the order of magnitude. Dozens of viruses infecting *Halorubrum* spp. have been reported from nine geographically distant hypersaline environments through culture-depend approach [3, 7, 11, 13]. During the screening of haloarchaeal viruses in Dingyuan Salt Mine using *Halorubrum* sp. strain LN27 and other haloarchaeal strains (Table S1) as the host strain, VOLN27B was isolated which can form a clear plaque on the lawn of strain LN27 (Figure 1). The diameter of the plaque strain LN27 agar plate was increased from 3 to 7 mm along with the extension of cultivation period. The transparency of the peripheral region was lower than that of the infectious center (Figure 1).

Viruses can be classified into two different categories based on their lifestyles: temperate and lytic [28]. Halovirus VOLN27B was isolated from an environmental sample (drill core) instead of induction from haloarchaea such as the halovirus SNJ1 [29]. Single-step growth curve of VOLN27B with a relatively high m.o.i. (m.o.i = 20) showed that the cell density did not decrease until 18 h p.i., and the virus titer in the supernatants obviously increased after 10 h p.i. and reached the highest at 21 h p.i. (Figure 2). The latent period of VOLN27B was about 10 h. The single-step growth curve profile of VOLN27B exhibiting an increase within 10 h p.i. followed by a sharp decrease, which was fairly similar to those of SH1 [30], HVTV-1 [18], and PH1 [31]. There are also some haloarchaeal viruses, e.g., HHPV-1 and HRPV-1, do not lead to cell lysis which just retard the growth of cells slightly [15, 32]. Unlike HHPV-1 or HRPV-1, reproduction of virus VOLN27B leads to cell lysis, suggesting that VOLN27B is a lytic virus, although viable cells still exist in the final cell suspension at the end of 36 h p.i. VOLN27B presenting different morphotypes does not lead to complete cell lysis.

Head-tailed haloarchaeal virus HF1 can infect strains from genera *Halobacterium* and *Haloferax*, while HF2 can infect strains from different species (*Halorubrum coriense* and *Hrr. saccharovorum*) in genus *Halorubrum* [33]. However, VOLN27B did not display a broad-host-range character even though a number of different species from genus *Halorubrum* and different genera have been used (Table S2).

VOLN27B presented an icosahedral head and a contractile tail (Figure 5(c)), which was reasonable for assigning VOLN27B to be a myovirus. The tailed icosahedral dsDNA viruses represented the most numerous archaeal virus group described thus far [10]. Even though we did not obtain any other strains which VOLN27B can infect which may attribute to the bias of approaches used in haloarchaea isolation for screening virus host, myoviruses and myophages can have broad host ranges typically [7, 34].

Pleomorphic haloviruses, e.g., HRPV-1, HHPV-1, HRPV11, and HRPV12, were sensitive to chloroform which caused four orders of magnitude loss of infectivity [3, 15, 32] due to the presence a membrane envelope. However, chloroform did not lead to order of magnitude loss of infectivity in VOLN27B (Figure 3(a)). It is consistent with the proposal that none of the head-tailed viruses are sensitive to chloroform so far [8].

Infectivity of VOLN27B decreased along with the decreasing of salinity (Figure 2(c)), and virus VOLN27B remained infectivity (10^2^ pfu/mL), which was very similar to the head-tailed halovirus phiN infecting *Halobacterium salinarum* [35]. Haloviruses will regained infectivity when the high salinity was restored, which was important for viral survival in natural habitats in which occasional salinity fluctuations were occurred [18]. In summary, we identified a new infectious myovirus, VOLN27B, from an underground salt crystal sample formed at the Cretaceous period. The virus stability, morphotype, and genomic type of this new halovirus have been determined which expands our knowledge on halovirus community from salt mine. Halovirus VOLN27B and *Halorubrum* sp. strain LN27 certainly provide a good virus-host system for probing the interaction between virus and its host.

## Data Availability

The 16S rRNA genes of Halorubrum sp. strains LN27, LN60, and LN72 were deposited in the GenBank/EMBL/DDBJ database under accession numbers MN829451.1, MN 826834.1, and MN 829452.1, respectively.

## Supplementary Materials

Supplementary 1 Table S1: strains used for screening halovirus.

Supplementary 2 Table S2: strains used for screening host range.

Supplementary 3 Figure S1: phylogenetic analysis of the host strain of VOLN27B along with other head-and-tail haloviruses based on 16S rRNA gene sequences using Neighbor-joining algorithm (Bootstrap method) in MEGA 10.0. Bootstrap Replications, 1000; Substitutions Model, Poisson model; Rates among Sites, Gamma Distributed; Gamma Parameter, 1; Gaps/Missing Data Treatment, Pairwise deletion. The host strain of halovirus HF1 is used as outgroup. Haloviruses and a provirus are shown on the right next to its host strain. Percentage support values are shown at branch points. Tree scale is indicated by the bar.

Supplementary 4 Figure S2: nucleic acid type of VOLN27B genome. Genomic DNA of VOLN27B (a), circular plasmid pBR322 (b), and linearized pBR322 at EcoR I site (c) were digested with DNase I (5 u), Exonuclease III (20 u), and Mung Bean Nuclease (4 u), respectively. The DNA molecular weight standards are shown on the left.
