# Supplementary material for: VOLN27B: A New Head-Tailed Halovirus Isolated from an Underground Salt Crystal and Infecting Halorubrum
Source: Archaea. 2021 Dec 14;2021:8271899. doi: 10.1155/2021/8271899 (PMC8727067; doi:10.1155/2021/8271899)
Supplement: Supplementary 1 — Table S1: strains used for screening halovirus. [file 8271899.f1.docx]

**Table S1** Strains used for screening halovirus

| **Strains** | **Accession number of 16S rRNA gene** | **Source** |
| --- | --- | --- |
| *Halorubrum* sp. LN27 | MN829451.1 | (1) |
| *Halorubrum*sp. LN60 | MN826834.1 | (1) |
| *Halorubrum*sp. LN72 | MN829452.1 | (1) |
| *Haloarcula hispanica* ATCC 33960 | NR_118849.1 | ATCC^a^ |
| *Haloferax*  *mediterranei* ATCC 33500 | NR_028212.1 | ATCC |
| *Halorubrum glutamatedens* ZY8 | NR_165753.1 | (2) |
| *Halobaculum roseum* D90 | NR_156820.1 | (3) |
| *Halococcus salsus* ZJ1 | MG097854.2 | (4) |
| *Halalkalicoccus subterraneus* GSM28 | NR_164956.1 | (5) |
| *Halobellus captivus* ZY21 | NR_165787.1 | (6) |

^a^Purchased from American Type Culture Collection.

Reference:

1. Chen L, Li F, Sun S, Xu Y, Chen S. 2019. Species diversity of culturable halophilic microorganisms isolated from Dingyuan salt mine, Anhui. Microbiology China 49:2186-2197 (In Chinese).

2. Xu, Y., Lv, J., Xie, C., Sun, S., Ke, L. and Chen, S. (2019) Halorubrum glutamatedens sp. nov., a Halophilic Archaeon Isolated from a Rock Salt. *Curr Microbiol*, **76**, 52-56.

3. Chen, S., Xu, Y., Liu, H.C., Yang, A.N. and Ke, L.X. (2017) Halobaculum roseum sp. nov., isolated from underground salt deposits. *Int J Syst Evol Microbiol*, **67**, 818-823.

4. Chen, S., Sun, S., Xu, Y. and Liu, H.C. (2018) Halococcus salsus sp. nov., a novel halophilic archaeon isolated from rock salt. *Int J Syst Evol Microbiol*, **68**, 3754-3759.

5. Chen, S., Xu, Y., Sun, S., Chen, F. and Liu, J. (2019) Halalkalicoccus subterraneus sp. nov., an extremely halophilic archaeon isolated from a subterranean halite deposit. *Antonie Van Leeuwenhoek*, **112**, 1067-1075.

6. Chen, S., Sun, S., Xu, Y., Chen, F. and Liu, J. (2020) Halobellus captivus sp. nov., an extremely halophilic archaeon isolated from a subterranean salt mine. *Antonie Van Leeuwenhoek*, **113**, 221-231.
