# Supplementary material for: VOLN27B: A New Head-Tailed Halovirus Isolated from an Underground Salt Crystal and Infecting Halorubrum
Source: Archaea. 2021 Dec 14;2021:8271899. doi: 10.1155/2021/8271899 (PMC8727067; doi:10.1155/2021/8271899)
Supplement: Supplementary 2 — Table S2: strains used for screening host range. [file 8271899.f2.docx]

**Table S2** Strains used for screening host-range

| Strains | Source |
| --- | --- |
| *Halorubrum aidingense* 31-hong(T) | CHL* |
| *Halorubrum* *aquaticum* CGMCC 1.6377(T) | CHL |
| *Halorubrum* *arcis* AJ201(T) | CHL |
| *Halorubrum* *californiense* SF3 213(T) | CHL |
| *Halorubrum* *chaoviator* Halo-G(T) | CHL |
| *Halorubrum* *depositum* Y78(T) | Our lab |
| *Halorubrum* *kocurii* JCM 14978(T) | CHL |
| *Halorubrum* *lacusprofundi* ATCC 49239(T) | CHL |
| *Halorubrum* *lipolyticum* 9-3(T) | CHL |
| *Halorubrum* *saccharovorum* JCM 8865(T) | CHL |
| *Halorubrum* *trueperi* Y73(T) | Our lab |
| *Halorubrum* *yunnanense* Q85(T) | Our lab |
| *Halopenitus* *malekzadehii* IBRC-M10418(T) | CHL |
| *Halopenitus* *persicus* DC30(T) | CHL |
| *Halalkalicoccus* *jeotgali* CGMCC 1.8490 (T) | CGMCC |
| *Halococcus* *saccharolyticus* CGMCC 1.6994 (T) | CGMCC |
| *Haloarchaeobius salinus* CGMCC 1.12232 (T) | CGMCC |
| *Haloferax* *denitrificans* CGMCC 1.2198 (T) | CGMCC |
| *Haloferax* *prahovense* CGMCC 1.8189 (T) | CGMCC |
| *Halostagnicola* *larsenii* CGMCC1.5338(T) | CGMCC |
| *Haloterrigena* *jeotgali* CGMCC 1.6228 (T) | CGMCC |
| *Haloparvum* *sedimenti* DYS4(T) | Our lab |
| *Natrinema* *pellirubrum* CGMCC 1.3708 (T) | CGMCC |
| *Natronomonas* *pharaonis* CGMCC 1.1965(T) | CGMCC |
| *Natrialba* *aegyptia* CGMCC 1.2639 (T) | CGMCC |

CHL*, strains donated from Prof. Henglin Cui (Jiangsu University, China); CGMCC, strains purchased from CGMCC (China General Microbiological Culture Collection Center).
