# Supplementary figures and images for: VOLN27B: A New Head-Tailed Halovirus Isolated from an Underground Salt Crystal and Infecting Halorubrum
Source: Archaea. 2021 Dec 14;2021:8271899. doi: 10.1155/2021/8271899 (PMC8727067; doi:10.1155/2021/8271899)

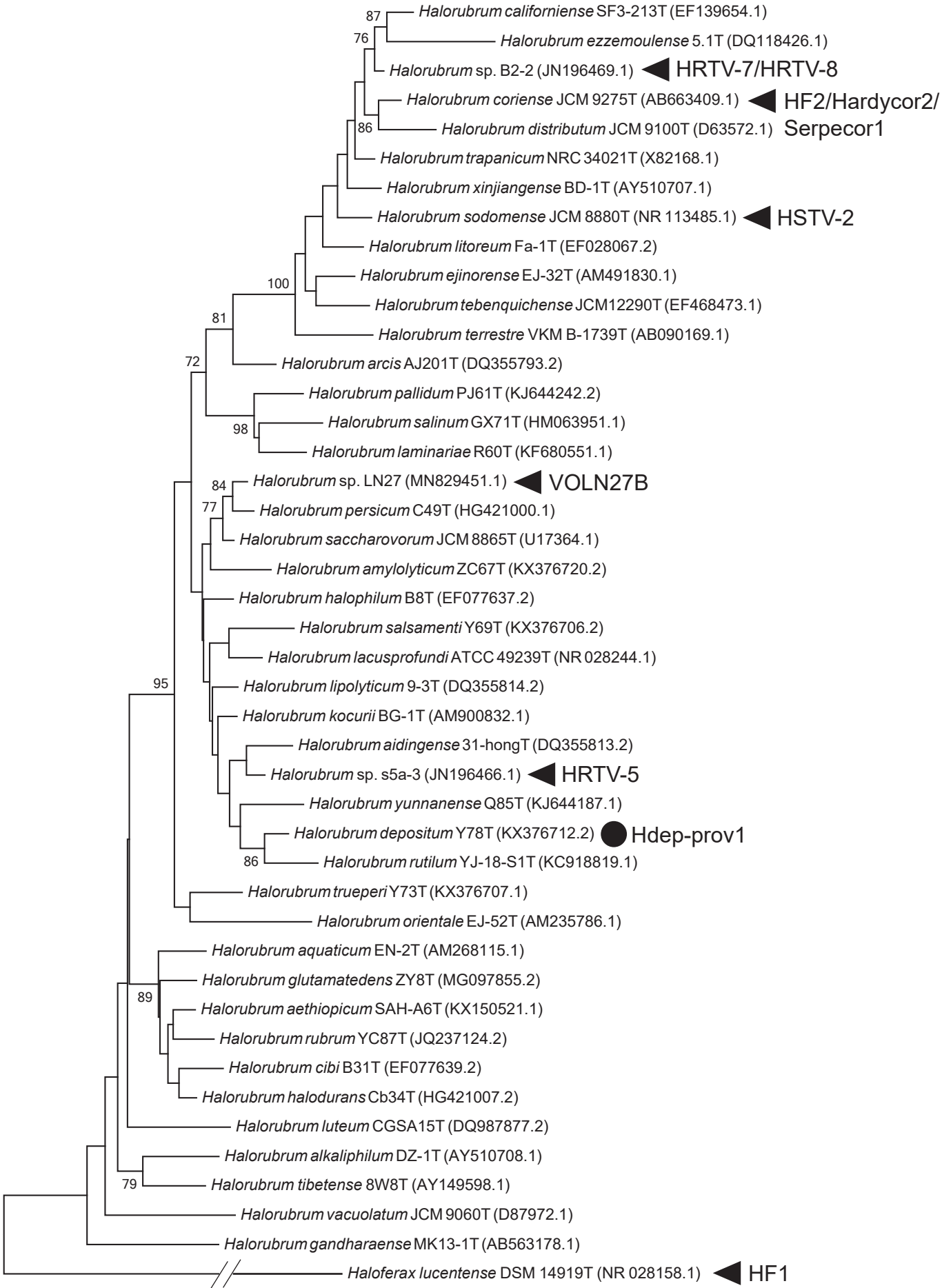

0.0100

Supplement: Supplementary 3 — Figure S1: phylogenetic analysis of the host strain of VOLN27B along with other head-and-tail haloviruses based on 16S rRNA gene sequences using Neighbor-joining algorithm (Bootstrap method) in MEGA 10.0. Bootstrap Replications, 1000; Substitutions Model, Poisson model; Rates among Sites, Gamma Distributed; Gamma Parameter, 1; Gaps/Missing Data Treatment, Pairwise deletion. The host strain of halovirus HF1 is used as outgroup. Haloviruses and a provirus are shown on the right next to its host strain. Percentage support values are shown at branch points. Tree scale is indicated by the bar. [file 8271899.f3.PDF]

**a**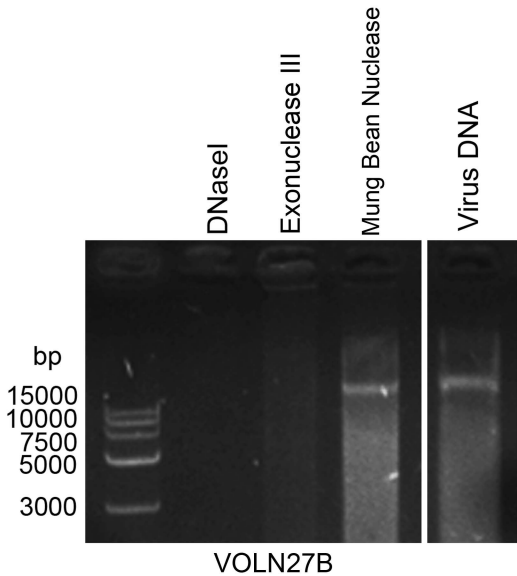**b**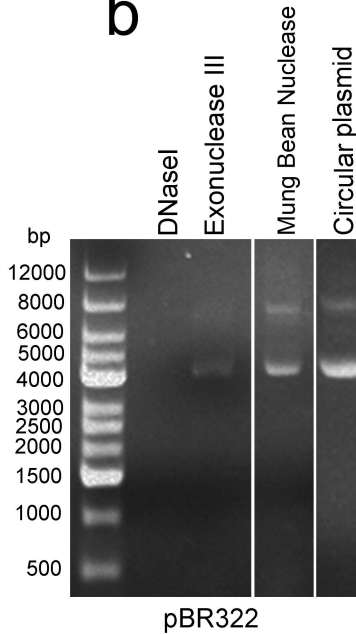**c**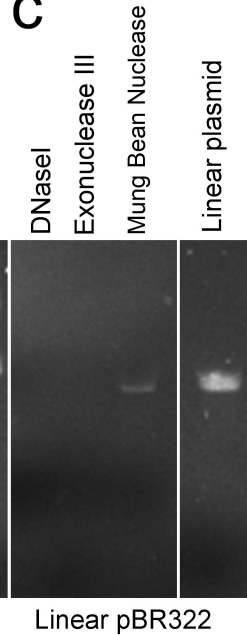

Supplement: Supplementary 4 — Figure S2: nucleic acid type of VOLN27B genome. Genomic DNA of VOLN27B (a), circular plasmid pBR322 (b), and linearized pBR322 at EcoR I site (c) were digested with DNase I (5 u), Exonuclease III (20 u), and Mung Bean Nuclease (4 u), respectively. The DNA molecular weight standards are shown on the left. [file 8271899.f4.pdf]
